# Screening, treatment initiation, and referral for substance use disorders

**DOI:** 10.1186/s13722-017-0083-z

**Published:** 2017-08-07

**Authors:** Steven L. Bernstein, Gail D’Onofrio

**Affiliations:** 1 Department of Emergency Medicine, Yale University School of Medicine, 464 Congress Ave., Suite 260, New Haven, CT 06519 USA; 2 Department of Health Policy, Yale School of Public Health, New Haven, CT USA; 3 Yale Cancer Center, New Haven, CT USA

**Keywords:** Brief intervention, Substance use, Screening, brief intervention, referral to treatment, Emergency medicine

## Abstract

Substance use remains a leading cause of preventable death globally. A model of intervention known as screening, brief intervention, and referral to treatment (SBIRT) was developed decades ago to facilitate time- and resource-sensitive interventions in acute care and outpatient settings. SBIRT, which includes a psychosocial intervention incorporating the principles of motivational interviewing, has been shown to be effective in reducing alcohol consumption and consequences in unhealthy drinkers both in primary care and emergency department settings. Subsequently, SBIRT for unhealthy alcohol use has been endorsed by governmental agencies and professional societies in multiple countries. Although most trials support the efficacy of SBIRT for unhealthy alcohol use (McQueen et al. in Cochrane Database Syst Rev 8, 2011; Kaner et al. in Cochrane Database Syst Rev 2, 2007; O’Donnell et al. in Alcohol Alcohol 49(1):66–78, 2014), results are heterogenous; negative studies exist. A newer approach to screening and intervention for substance use can incorporate initiation of medication management at the index visit, for individuals willing to do so, and for providers and healthcare systems that are appropriately trained and resourced. Our group has conducted two successful trials of an approach we call screening, treatment initiation, and referral (STIR). In one trial, initiation of nicotine pharmacotherapy coupled with screening and brief counseling in adult smokers resulted in sustained biochemically confirmed abstinence. In a second trial, initiation of buprenorphine for opioid dependent individuals resulted in greater engagement in treatment at 30 days and greater self-reported abstinence. STIR may offer a new, clinically effective approach to the treatment of substance use in clinical care settings.

High risk health behaviors, including substance use, remain leading causes of preventable death globally. A model of intervention known as screening, brief intervention, and referral to treatment (SBIRT) was developed 20 years ago to facilitate time- and resource-sensitive interventions in acute care and outpatient settings. SBIRT, which includes a psychosocial intervention incorporating the principles of motivational interviewing, has been shown to be effective in reducing alcohol consumption and consequences in unhealthy drinkers both in primary care and emergency department settings (ED). Subsequently, SBIRT for unhealthy alcohol use has been endorsed by governmental agencies and professional societies in multiple countries, including the World Health Organization, American College of Emergency Physicians, the Committee on Trauma of the American College of Surgery, the Substance Abuse and Mental Health Services Administration and the United States Preventive Services Task Force, and the College of Family Physicians of Canada.

SBIRT appears to be an effective strategy for unhealthy alcohol use, although results from clinical trials are mixed, and treatment effects are modest. An additional challenge is that the term “SBIRT” has since been applied to other substances, such as tobacco and opioids. A third concern is that the effect of the site of care, reason for visit, interventionists’ training, or subject’s motivation to change behavior on SBIRT’s efficacy has not been adequately studied. A final concern is that the “brief intervention” component of SBIRT is restricted to behavioral treatments, and omits the many FDA-approved, efficacious pharmacotherapies for the treatment of alcohol, tobacco, and opioid use disorder.

Three recently published trials of brief intervention for individuals with substance use disorders found no benefit. Two were conducted in primary care settings [4, 5], and one in the ED [6]. Similarly, most studies of behavioral treatment for substance use or tobacco dependence in ED populations have been negative [7, 8].

To address some of the shortcomings of SBIRT, we have extended the model by adding the initiation of pharmacotherapy to the therapeutic options of the interventionist, at the index visit. Two recent studies using this model, performed by our group, were successful in decreasing tobacco and opioid use, respectively. This model, which we call screening, treatment initiation, and referral (STIR), represents a paradigm shift in the identification and treatment of individuals with substance use disorders.

We review two recent studies that support the use of STIR, and make recommendations. Both studies were clinical trials conducted in the ED [9, 10].

Bernstein et al. [9] randomized 778 adult smokers in an urban ED to a control or intervention group. Eligible subjects were adults presenting to the ED for any reason. The intervention consisting of an abbreviated version of a motivational interview, a referral faxed to the state smokers’ quitline, a phone call 2–3 days after enrollment, and the provision of six weeks of nicotine patches and gum. The first dose of patch or gum was ED-initiated. Patients in both groups received a brochure promoting the quitline and providing information about the health risks of smoking. At three months, the biochemically confirmed abstinence rate in the intervention group, 12.2% was significantly greater than the control group 4.9% (*P* < 0.001).

D’Onofrio et al. [10] randomized 329 adults with opioid dependence presenting to the ED (for any condition) to one of three groups: (1) a referral to community-based opioid addiction treatment services; (2) a traditional SBIRT intervention, consisting of a BNI with a facilitated, direct referral to addiction treatment services, or (3) a BNI, ED-initiated buprenorphine and referral to primary care to 10 weeks of continued medical management. The primary outcome for the study was engagement in addiction services treatment at 30 days after randomization.

The STIR strategy, ED-initiated buprenorphine, significantly increased patients’ engagement in addiction treatment at 30 days compared with the SBIRT and referral groups: 78, 45, and 37%, respectively (*P* < 0.001) and decreased self-reported illicit drug use. In addition, patients in the STIR group were less likely to be enrolled in inpatient addiction services at 30 days, compared to those in the SBIRT or referral groups. There were no differences in rates of negative urine tests.

In both trials, patients were screened for eligibility by trained research assistants. All were adults age 18 years or older, presenting to the ED for any medical or surgical condition; patients with primarily behavioral disorders (e.g. acute psychosis) were excluded. BNIs, conducted by the research assistants, typically lasted 10–15 min. BNIs adapted principles of motivational interviewing, and had four components: permission to discuss substance use, feedback on the health consequences of ongoing substance use, motivational enhancement, and negotiation and advice. In both studies, two-thirds of eligible subjects consented to enroll.

The standard SBIRT model focuses on alcohol. It consists of identifying individuals with risky levels of drinking, and engaging with them using techniques adapted from motivational interviewing or motivational enhancement. For individuals who meet criteria for alcohol use disorder, referral to follow-up in a treatment program is recommended. The referral may consist of giving the individual the contact information for treatment programs, or may involve phoning or electronic referral for a more definitive linkage to care. Printed materials may be provided. Interventions are generally delivered by clinicians, but may also be delivered by other healthcare personnel, or trained lay providers. Initiation of pharmacotherapy is not part of this traditional approach.

STIR adds pharmacotherapy to SBIRT, and may be efficacious for several reasons:

- *Salience of the acute care visit* The ED visit has often been described as a “teachable moment,” in which an individual presenting with an acute illness or injury caused by a risky health behavior may be amenable to initiate a change in that behavior. Numerous theories of behavior change have been offered as conceptual models to explain the teachable moment phenomenon [11]. In our work we enroll patients who present to the ED with any condition, irrespective of whether it is caused or exacerbated by the substance use. In general, it is possible to discuss how their substance use may adversely affect treatment of the acute condition.
- *Additive or synergistic effects of pharmacotherapy* In the treatment of tobacco dependence, a rich literature describes the enhanced efficacy of combination NRT compared to monotherapy, and medication and counseling together as more effective than either alone [12].
- *Lowering patients’ perceived barriers to medication use* Substance users often have diminished access to primary care [13]. Beginning medication during the ED visit allows providers to educate patients on the benefits of medications such as opioid agonists or nicotine replacement therapies, answer questions, and demonstrate application of the nicotine patch.
- *Physicians and providers are comfortable prescribing medications* Medical school and residencies traditionally offered limited training in the behavioral treatment of substance use [14], although this is evolving. STIR incorporates behavioral interventions, but allows physicians to use their traditional skills in medication management. For the physician unwilling or unable to provide a behavioral intervention, it is reasonable to begin pharmacotherapy. That said, additional training in the medication-assisted treatment of opioid and alcohol use disorders is likely to be needed.

Implementing a program of STIR requires attention to important operational details. These include:

## *Screening and assessment for eligibility for specific medications*

 Protocols for screening, medication eligibility, prescribing, management of withdrawal, and follow-up are needed. This includes partnering with community providers for ongoing care. That said, if STIR should occur in a primary care setting, that setting itself may become the site of follow-up care, in which case the “referral” is part of normal clinic workflow. Whether the clinical setting is primary care or ED, protocols should be sensitive to the need for efficiency and timeliness of care.

Of note, “assessment” may also entail determination of the patient’s willingness to change, although newer paradigms of screening for tobacco use would make treatment the default approach for individuals with identified substance use disorders [15]. The clinical efficacy of making treatment the default for other substances of abuse remains to be determined.

## *Delivery of the BNI*

 In our trials, the BNI was delivered by trained, nonclinical research personnel. BNIs were audiotaped and reviewed biweekly by research assistants and a psychologist. This model is not easily replicable, but physicians from diverse specialties can be trained to deliver BNIs [16].

## *Pharmacologic treatment*

 Our STIR trials focused on tobacco and opioids, two substances that are widely used and misused. Each has multiple effective, approved medications to treat dependence, with evidence to support reduction in subsequent healthcare utilization [17]. For tobacco, there are seven approved medications: nicotine patch, gum, lozenge, nasal spray, and inhaler, and varenicline and bupropion. For opioid dependence, both methadone and buprenorphine are approved, in addition to the use of naloxone for acute overdoses. In primary care settings, it may be appropriate to consider initiation of naltrexone for alcohol use disorders as well.

Additional scientific and clinical challenges remain. The efficacy of STIR should be confirmed with additional trials in other care settings. Whether the counseling component of STIR can be delivered effectively and reliably by the treating clinician needs to be assessed. In addition, STIR’s efficacy should be assessed for other substances that have effective, agency-approved pharmacotherapy. A good example is alcohol, for which medications such as naltrexone has shown efficacy in reducing use and craving.

Finally, in the United States, new models of care delivery, and enhanced insurance coverage for medication, spurred by the Affordable Care Act, may promote the implementation of STIR. Healthcare systems are increasingly incentivized to manage the health of populations. Integrating STIR into clinical workflows facilitates the treatment of substance use.

ED-based interventions for cigarette smoking and opioid dependence demonstrate that screening and treatment initiation, combining brief counseling, pharmacotherapy, and referral, promote abstinence and increased engagement in treatment. In the ED, STIR has the potential to narrow the gap between services needed and treatment. Its utility in other care settings deserves study. Given the prevalence of substance use, and its associated mortality, morbidity, and cost, we suggest that the moment is ripe for new models of treatment that will facilitate the identification and treatment of individuals with substance use disorders.
